# Volatile Organic Compounds from *Centaurium erythraea* Rafn (Croatia) and the Antimicrobial Potential of Its Essential Oil

**DOI:** 10.3390/molecules17022058

**Published:** 2012-02-20

**Authors:** Igor Jerković, Dajana Gašo-Sokač, Hrvoje Pavlović, Zvonimir Marijanović, Mirko Gugić, Ivana Petrović, Spomenka Kovač

**Affiliations:** 1 Department of Organic Chemistry, Faculty of Chemistry & Technology, University of Split, N. Tesle 10/V, HR-21000 Split, Croatia; 2 Faculty of Food Technology, University J. J. Strossmayer in Osijek, Kuhačeva 20, HR-31000 Osijek, Croatia; Email: dajana.gaso@ptfos.hr (D.G.-S.); Spomenka.Kovac@ptfos.hr (S.K.); 3 Department of Chemistry, University J. J. Strossmayer in Osijek, Kuhačeva 20, HR-31000 Osijek, Croatia; 4 Department of Food Technology, Polytechnic “Marko Marulić” in Knin, P. Krešimira IV 30, HR-22300 Knin, Croatia; Email: zmarijanovic@veleknin.hr (Z.M.); mirko.gugic@veleknin.hr (M.G.)

**Keywords:** *Centaurium erythraea* Rafn, headspace, essential oil, microcolumn fractionation, gas chromatography and mass spectrometry, disk diffusion method

## Abstract

GC and MS were used for the analysis of Croatian *Centaurium erythraea* Rafn essential oil (obtained by hydrodistillation) and headspace (applying headspace solid-phase microextraction). The headspace contained numerous monoterpene hydrocarbons (the major ones were terpinene-4-ol, methone, *p*-cymene, γ-terpinene and limonene). Oxygenated monoterpenes were present in the headspace and oil, while 1,8-cineole, bornyl acetate and verbenone were present only in the headspace. High headspace percentages of toluene and naphthalene were found, followed by hemimellitene. Lot of similarities were observed with Serbian *C.erythraea* oil [neophytadiene (1.4%), thymol (2.6%), carvacrol (6.1%) and hexadecanoic acid (5.7%)], but different features were also noted such as the presence of menthol, menthone and phytone. The oil fractionation enabled identification of other minor compounds not found in total oil such as norisoprenoides, alk-1-enes or chromolaenin. The essential oil showed antimicrobial potential on *Escherichia coli*, *Salmonella enteritidis*, *Staphylococcus aureus* and *Bacillus cereus*. On the other hand, no antibacterial activity of the oil was observed on *Pseudomonas fluorescens* and *Lysteria monocytogenes*.

## 1. Introduction

Small centaury, *Centaurium erythraea* Rafn (*Gentianaceae*) is traditional medicinal plant in Croatia. The use of *C. erythraea* in traditional medicine has been described in the pharmacopoeia of 23 different countries. It has been used for the treatment of asthma, eczema, rheumatism, wounds and sores, as well to reduce gastrointestinal smooth muscle spasm and digestive disorders (loss of appetite, stomach discomfort and indigestion) [1]. Previous phytochemical studies [2,3,4,5] on *C. erythraea* revealed the presence of a variety of plant secondary metabolites, including centauroside, centapiricin, flavonoids, gentiopicrin, gentiopicroside, isocumarin, phenolic acids and their derivatives, swertiamarin, terpenoids and xanthones. Many of these compounds are known to exhibit important biological (antimicrobial, antimutagenic and antioxidative) activities. Investigations showed that *C. erythraea* (lyophilised infusion) is an effective antioxidant with the ability to scavenge superoxide radical and noncompetitively inhibit xanthine oxidase [6,7]. Anti-inflammatory and antipyretic effects of an aqueous extract of the plant have also been observed experimentally in rats [8]. Antibacterial activity of *C. erythraea* infusion was tested on several bacterial species, and *Bacillus megaterium* exhibited the highest sensitivity, while *Escherichiacoli* and *Staphylococcus aureus* were not sensitive to the infusion [9].

*C. erythraea* has been the subject of several physiochemical investigations, but the chemical composition of its essential oil was studied only recently in Serbia [10], while there is no data on its headspace composition. In the literature, there have also been no attempts to investigate the antibacterial effect of the essential oil. Therefore, the aim of the present study was to investigate the phytochemical composition of volatile organic compounds (VOCs) of Croatian *C. erythraea* Rafn (including headspace), and also to establish the antimicrobial potential of its essential oil on selected Gram-positive and Gram-negative bacterial species. In order to obtain more detail volatiles chemical composition a two-way approach was used: headspace solid-phase microextraction (HS-SPME) and hydrodistillation (HD). Due to the expected complex oil composition, the fractionation into non-polar and polar fractions was performed by silicagel microcolumn chromatography. All isolates were analysed by gas chromatography and mass spectrometry (GC, GC-MS).

## 2. Results and Discussion

Hydrodistillation (HD) of *C. erythraea* aerial parts gave a yellow oil (yield 0.02%). The plant VOCs present in the headspace (obtained by HS-SPME) and essential oil (obtained by HD) were analysed by GC and GC-MS. The oil was further fractionated by silicagel microcolumn chromatography (yielding polar and non-polar fractions) in order to avoid potential overlapped GC peaks. In addition, the isolated oil was tested for unlocking its antimicrobial potential against selected Gram positive and Gram negative cultures.

### 2.1. The Headspace *C. erythraea* VOC Composition

Two fibers (PDMS/DVB and DVB/CAR/PDMS) were selected for HS-SPME after preliminary research with respect to overall number of isolated compounds. Both fibers showed qualitatively similar chemical profiles of the extracted compounds, but individual compound percentages varied (Table 1). A total of 52 VOCs were identified and reported for the first time in *C. erythraea* headspace.

**Table 1:** The headspace volatiles of *C. erythraea* obtained by HS-SPME with the fibers: **A**–PDMS/DVB and **B**–DVB/CAR/PDMS.

No.	Compound	RIHP-5MS	RIHP-FFAP	Area percentage (%)A B	
1.	2-Methylpentane ^*^	<900	/	0.5	0.6
2.	3-Methylpentane ^*^	<900	/	1.1	1.7
3.	Hexane	<900	< 900	0.6	2.0
4.	Ethyl acetate	<900	901	-	0.8
5.	Methylcyclopentane	<900	/	1.7	1.9
6.	Cyclohexane	<900	/	0.5	0.8
7.	Pentanal	<900	/	-	0.8
8.	Toluene	<900	1067	4.5	18.0
9.	Hexanal	<900	1105	1.5	4.8
10.	1,4-Dimethylbenzene ^*^	<900	/	-	0.4
11.	Heptanal	905	/	-	0.4
12.	α-Thujene	933	/	-	0.6
13.	α-Pinene	941	1040	-	1.0
14.	Propylbenzene (Isocumene)	960	/	-	0.6
15.	*m*-Ethyltoluene ^*^	968	/	0.9	1.7
16.	1,2,4-Trimethylbenzene (Pseudocumene)	976	/	0.9	1.0
17.	Sabinene	982	1140	0.6	1.3
18.	β-Pinene	984	1130	-	1.1
19.	*o*-Ethyltoluene ^*^	987	/	0.8	0.8
20.	2-Pentylfuran	996	1249	1.0	2.4
21.	1,2,3-trimethylbenzene (Hemimellitene)	1002	/	1.8	2.5
22.	*p-*Cymene	1032	1293	2.8	3.5
23.	Limonene	1036	1220	1.5	1.8
24.	1,8-Cineole	1039	1231	1.1	0.9
25.	γ-Terpinene	1066	/	2.2	2.2
26.	α-Terpinolene	1094	/	-	0.5
27.	Undecane	1100	1100	-	0.7
28.	Linalool	1107	1560	2.8	0.9
29.	Nonanal	1109	/	2.9	1.8
30.	β-Thujone	1112	1450	2.8	1.2
31.	Camphor	1152	1548	5.1	2.0
32.	Menthone	1162	1490	6.1	4.3
33.	Isomenthone	1172	1519	0.9	1.6
34.	Menthol	1183	1658	2.9	6.7
35.	Terpinen-4-ol	1186	1623	10.3	6.9
36.	Naphthalene	1190	/	8.2	1.8
37.	α-Terpineol	1198	1717	1.5	0.6
38.	Decanal	1211	1518	2.3	1.1
39.	Verbenone	1217	/	0.7	0.4
40.	Bornyl acetate	1295	1601	0.9	0.4
41.	Safrole	1295	/	2.7	-
42.	Menthyl acetate	1299	/	1.6	0.9
43.	Tridecane	1300	1300	0.9	1.0
44.	α-Copaene	1382	1510	2.0	0.8
45.	(*E*)-β-Damascenone	1390	1848	1.1	-
46.	Tetradecane	1400	1400	3.2	1.4
47.	Longifolene	1409	1592	1.0	1.6
48.	β-Caryophyllene	1425	1618	3.7	1.6
49.	*trans*-β-Farnesene	1464	1677	2.5	0.7
50.	Hexadecane	1600	1600	1.8	1.4
51.	Heptadecane	1700	1700	1.0	-
52.	Nonadecane	1900	1900	0.8	-
Total identified (%)				93.7	93.9

RI = retention indices on HP-5MS and HP-FFAP columns; **A** = solvent-free HS-SPME with the fiber PDMS/DVB; **B** = solvent-free HS-SPME with the fiber DVB/CARPDMS; - = compound not found; / = compound not identified on the column; * – correct isomer not identified.

Aliphatic and aromatic hydrocarbons were abundant, particularly toluene (4.5%; 18.0%) and naphthalene (8.2%; 1.8%) followed by *o*- and *m*-ethyltoluene (0.8–1.7%). The trimethylbenzenes pseudocumene (0.9%; 1.0%) and hemimellitene (1.8%; 2.5%) were also present, but were not found in the oil composition (Table 2). Although benzene derivatives are usually considered as compounds of possible anthropogenic origin, new results indicate possible other pathways of their natural biogenesis. Namely, the emission of toluene from different plants was observed, although no biochemical pathway for toluene production is known [11]. Trace naphthalene amounts are produced by magnolias and flower extracts from gynoecia of five taxa (*Magnolia denudata, Magnolia liliiflora, Magnolia tomentosa, Magnolia praecocissima* var. *praecocissima* and var. *borealis*) contained naphthalene as main component [12]. Furthermore, Formosan subterranean termite [13] and some strains of the fungus *Muscodor albus* naturally produce naphthalene, while *Muscodor vitigenus* produces naphthalene almost exclusively [14]. Therefore natural origin of the benzene derivatives found in *C. erythraea* oil could be similar, and they can be excluded as pollutants since the plant was collected from ecologically pure area. Aliphatic hydrocarbons and carbonyls up to C18 were present as minor constituents (probably originated from fatty acids catabolism) and those up to C6 were only found in *C. erythraea* headspace (most likely due to high volatility and solvent delay applied for the oil GC analysis). 

**Table 2:** The essential oil composition of *C. erythraea* (**C**) and its fractions: **D**–non-polar fraction and **E**–polar fraction.

No.	Compound	RIHP-5MS	RIHP-FFAP	Peak area (%)C D E		
1.	Ethyl acetate	<900	901	-	-	1.3
2.	Hexanal	<900	1105	-	-	1.1
3.	4-Methyloctane ^*^	<900	/	-	0.3	-
4.	1,4-Dimethylbenzene ^*^	<900	/	-	0.2	-
5.	Nonane	900	900	-	8.6	0.2
6.	Undecane	1100	1100	-	0.6	-
7.	Linalool	1107	1560	3.0	-	3.9
8.	Nonanal	1109	/	0.2	-	0.5
9.	β-Thujone	1112	1450	0.8	-	1.1
10.	Camphor	1152	1548	1.5	-	1.8
11.	Menthone	1162	1490	2.5	-	3.3
12.	Isomenthone	1172	1519	0.3	-	0.9
13.	Borneol	1175	1723	1.4	-	1.6
14.	Menthol	1183	1658	7.0	-	8.8
15.	Terpinen-4-ol	1186	1623	1.2	-	1.5
16.	α-Terpineol	1198	1717	1.1	-	1.4
17.	Methyl chavicol (Estragole)	1205	1697	2.6	1.1	0.9
18.	Decanal	1211	1518	0.1	-	0.3
19.	Pulegone	1248	1677	0.2	-	0.8
20.	Carvone	1252	1767	1.5	-	2.0
21.	Piperitone	1263	1761	0.7	-	0.3
22.	Geraniol	1264	1865	0.2	-	-
23.	Bornyl acetate	1295	1601	0.7	-	0.9
24.	*trans-*Anethole	1293	1860	3.6	1.5	1.0
25.	Menthyl acetate	1299	/	0.3	-	0.7
26.	Thymol	1307	2152	2.6	-	5.8
27.	Carvacrol	1318	2189	6.1	-	13.8
28.	*trans*, *trans*-Deca-2,4-dienal	1325	1839	0.2	-	-
29.	α-Copaene	1382	1510	0.3	0.6	-
30.	(*E*)-β-Damascenone	1390	1848	2.3		2.8
31.	Tetradec-1-ene	1397	/	-	0.5	-
32.	Tetradecane	1400	1400	-	0.3	-
33.	Longifolene	1409	1592	-	0.5	-
34.	Methyleugenol	1414	/	0.3	-	-
35.	β-Caryophyllene	1425	1618	0.9	1.1	-
36.	α-Humulene	1460	1691	-	0.5	-
37.	Geranyl acetone	1461	1875	1.3	-	0.7
38.	*trans*-β-Farnesene	1464	1677	1.3	1.0	-
39.	α-Amorphene	1483	1707	0.2	0.5	-
40.	Ar-curcumene	1490	1791	0.2	0.7	-
41.	(*E*)-β-Ionone	1493	1958	1.4	-	2.0
42.	α-Selinene	1500	1747	-	0.8	-
43.	α-Muurolene	1506	1745	0.3	-	-
44.	Pentadecane	1500	/	-	1.1	-
45.	β-Bisabolene	1515	1743	0.2	0.7	-
46.	γ-Cadinene	1521	1814	0.3	-	-
47.	δ-Cadinene	1531	1777	0.8	1.9	-
48.	Myristicin	1533	2271	-	-	0.3
49.	α-Calacorene	1551	1938	0.3	0.8	-
50.	Spathulenol	1586	2088	1.3	-	0.9
51.	Caryophyllene oxide	1589	1998	0.9	-	1.7
52.	Dodecanoic acid	1594	/	1.0	-	-
53.	Hexadec-1-ene	1598	/	-	1.7	-
54.	Viridiflorol	1599	2059	0.8	-	1.1
55.	Hexadecane	1600	1600	-	1.0	-
56.	α-Cadinol	1665	/	0.7	-	1.6
57.	Ethyldibenzothiophene (isomer I) ^*^	1682	/	0.7	-	-
58.	Acorenone B	1696	/	0.9	-	1.2
59.	Heptadecane	1700	1700	0.3	1.1	-
60.	Farnesol ^*^	1710	/	0.5	-	0.6
61.	Pentadecanal	1723	/	0.5	-	0.8
62.	Chromolaenin	1727	/	-	2.1	-
63.	Ethyldibenzothiophene (isomer II) ^*^	1729	/	-	0.6	-
64.	Ethyldibenzothiophene (isomer III) ^*^	1734	/	0.2	1.5	-
65.	Aristolone	1767	/	0.4	-	0.8
66.	Tetradecanoic acid	1791	/	1.5	2.3	0.7
67.	Octadec-1-ene	1799	/		2.6	-
68.	Octadecane	1800	1800	0.2	1.1	-
69.	Neophytadiene	1849	1925	1.4	4.5	-
70.	Hexahydroxyfarnesyl acetone (Phytone)	1857	2082	4.0	-	6.6
71.	Pentadecanoic acid	1896	/	0.2	-	-
72.	Nonadecane	1900	1900	0.7	4.6	-
73.	(*E*,*E*)-Farnesyl acetone	1928		0.9	-	1.5
74.	Methyl hexadecanoate	1937	/	0.3	-	0.7
75.	Isophytol	1960	/	0.3	-	0.2
76.	Hexadecanoic acid	1963	/	5.7	2.2	6.8
77.	Eicos-3-ene ^*^	1998	/	-	2.3	-
78.	Eicosane	2000	2000	-	1.5	-
79.	Heneicosane	2100	2100	2.2	8.7	-
80.	Phytol ^*^	2135	/	1.9	-	-
81.	Linoleic acid	2165	/	3.9	-	5.3
82.	Docosane	2200	2200	0.5	2.6	-
83.	Tricosane	2300	2300	6.8	25.7	-
84.	Tetracosane	2400	2400	1.3	4.7	-
85.	3-Methylbutyl 3-methylbutanoate	/	1306	0.2	-	-
86.	α-Thujone	/	1469	0.3	-	0.6
87.	Dihydroedulan II	/	1514	0.3	-	-
88.	Dihydroedulan I	/	1544	0.5	-	-
89.	Cuminal	/	1815	0.2	-	0.3
Total identified (%)				89.4	94.1	91.1

RI = retention indices on HP-5MS and HP-FFAP columns; **C** = total essential oil; **D** = non-polar fraction of the oil; **E** = polar fraction of the oil; - = compound not found; / = compound not identified on the column; * – correct isomer not identified.

Contrary to the oil chemical composition, the headspace contained abundant monoterpene hydrocarbons, the major ones being *p*-cymene (2.8%; 3.5%), γ-terpinene (2.2%; 2.2%) and limonene (1.5%; 1.8%). Several were only found in the headspace with low percentages (α-pinene, sabinene, β-pinene, *p*-cymene, limonene, γ-terpinene and α-terpinolene). Oxygenated monoterpenes were generally present in the headspace and essential oil (although with different abundances) and the major ones in the headspace were terpinene-4-ol (10.3%; 6.9%), menthone (6.1%; 4.3%), linalool (2.8%; 0.9%), β-thujone (2.8%; 1.2%), camphor (5.1%; 2.0%), isomenthone (0.9%; 1.6%), menthol (2.9%; 6.7%) and menthyl acetate (1.6%; 0.9%). Only few oxygenated monoterpenes were exclusively found in the headspace: 1,8-cineole, bornyl acetate and verbenone. β-Caryophyllene (3.7%; 1.6%), *trans*-β-farnesene (2.5%; 0.7%), α-copaene (2.0%; 0.8%) and longifolene (1.0%; 1.6%) were identified among sesquiterpenes with higher percentages in comparison to the oil, but great number of other essential oil sesquiterpenes (Table 2) were not found by HS-SPME.

### 2.2. *C. erythraea* Essential Oil Composition

A total of 89 compounds were identified in the essential oil of *C. erythraea* Rafn (Table 2). In comparison with the sole previous report on this oil from Serbia [10], the overall number of identified compounds seems moderate, but it should be emphasized that the previously published oil composition predominated (*ca.* 50%) with the compounds in traces (<0.05%). This research was focused on detailed determination of non-trace compounds of the oil including the results of the oil fractionation to non-polar and polar fractions.

Total essential oil contained a low abundance of oxygenated monoterpenes, the major ones being menthol (7.0%), linalool (3.0%), borneol (1.4%) and methone (2.5%). Menthol and menthone were not found in Serbian oil (only traces of isomenthol were detected) as well as β-thujone (0.8%). Borneol (1.4%) and camphor (1.5%) were identified in Croatian oil, while only traces were reported in Serbian oil. Monoterpene phenols thymol (2.6%) and carvacrol (6.1%) were interesting features also reported among the major constituents in Serbian oil (thymol 7.9% and carvacrol 4.2%). Although these phenols were present in the oil, they were not identified in the headspace. On the contrary, their biosynthetic precursors γ-terpinene and *p*-cymene were found in the headspace, but not in the oil.

Neophytadiene (10.1%) was major compound of Serbian oil, while in this research it amounted to 1.4%. Neophytadiene is presumably a chlorophyll metabolite [15]. Chlorophyll chlorin rings can be constructed from several different side chains, usually including the long diterpene alcohol phytol (found in the oil at 1.9%). Isophytol (0.3% in the oil) is a phytol isomer and isomerisation of isophytol to phytol in plant leaf waxes is reported [16]. Oxidation of the phytol moiety of chlorophyll could lead, among others, to the methylated long chain fatty acid ketone hexahydroxyfarnesyl acetone (6,10,14-trimethylpentadecan-2-one, phytone) that was found in the oil at 4.0%. The fourth major compound of Serbian oil was hexadecanoic acid (4.9%), while Croatian oil contained 5.7%, followed by linoleic acid (3.9%) and tetradecanoic acid (1.5%). High-molecular aliphatic hydrocarbons were present, particularly tricosane (6.8%). Their abundance was higher in comparison with Serbian oil. Sesquiterpenes were also found with minor percentages; the major ones were caryophyllene oxide (0.9%) and δ-cadinene (0.8%). As was expected, high-molecular compounds of the essential oil were not identified in the headspace due to low volatility.

**Figure 1:**
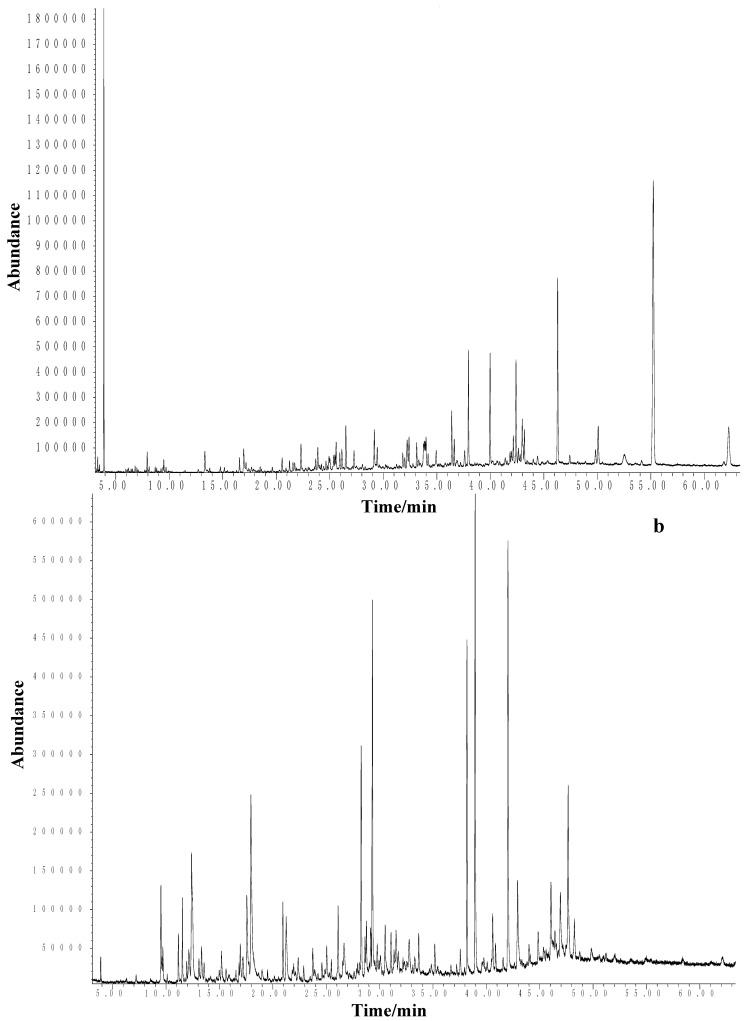
Representative TICs on HP-5MS column: (**a**) non-polar fraction; (**b**) polar fraction.

Silicagel microcolumn fractionation enabled a more detailed analysis by the oil separation into non-polar and polar compounds. As was expected from total oil composition, the fraction of non-polar compounds (Figure 1a) dominated by higher aliphatic hydrocarbons such as tricosane (25.7%), heneicosane (8.7%), nonadecane (4.6%) and others. However, the pentane fraction revealed the presence of low-molecular aliphatic hydrocarbons (overlapped in total oil), and nonane (8.6%) was the major one. In addition, alk-1-enes were only found in this fraction, including hexadec-1-ene (1.7%), octadec-1-ene (2.6%) and tetradec-1-ene (0.5%) as well as eicos-3-ene (2.3%). According to their chain length and positions of double bonds they are derived from corresponding fatty acids [17]. 

It is possible that some of the hydrocarbons found were hydrodistillation artifacts. The plants were collected from an ecologically pure area and the possibility of contamination is thus excluded. Natural existence of hydrocarbons in plants is also known. Many higher members of the *n*-alkanes, *n*-alkan-1-ols, *n*-alkanals, *n*-alkanoic acids and *n*-alkyl esters were identified in cuticular waxes [18].

The variety of identified sesquiterpene hydrocarbons in the non-polar fraction was mainly composed of δ-cadinene (1.9%), β-caryophyllene (1.1%) and *trans*-β-farnesene (1.0%). Their percentages were higher in comparison with the oil, and several were only identified in this fraction, and not in the oil. The rather unusual monooxygenated compound chromolaenin (2.1%), with a cadinane structure and previously reported in the essential oils of *Baccharis salicifolia*, *B. latifolia* and *B. dracunculifolia* [19], was also only detected in the pentane fraction. 

The oil polar fraction (Figure 1b) contained a variety of oxygen-containing monoterpenes (as expected with higher percentages in comparison to the oil), and the major ones were: menthol (8.8%), menthone (3.3%), linalool (3.9%), borneol (1.6%) and *trans*-anethole (1.0%). Thymol (5.8%) and carvacrol (13.8%) were among the most widespread compounds of this fraction. Oxygenated sesquiterpenes were principally composed of α-cadinol (1.6%), caryophyllene oxide (1.7%), acorenone B (1.2%) and viridiflorol (1.1%). Phytone (6.6%) was the most abundant chlorophyll-related compound. The diethyl ether fraction contributed to the identification of norisoprenoides [(*E*)-β-damascenone (2.8%) and (*E*)-β-ionone (2.0%)], a group of compounds exclusively present in the pentane fraction. Palmitic acid and linoleic acid were the main fatty acids in the fraction. 

### 2.3. Unlocking Antimicrobial Potential of the Essential Oil

The essential oil of *C. erythraea* showed different antimicrobial potential towards the bacterial species tested. In general, Gram-negative bacteria (*E. coli* and *S. enteritidis*) were the most sensitive species to the oil, while there was no effect to *P. fluorescens* growth. Ampicilin (10 μg), norfloxacin (10 μg), ofloxacin (5 μg) and tetracycline (30.5 μg) were used as positive reference standards to determine the sensitivity of bacterial strains tested. Although applied antibiotics possessed in general strongest antibacterial activity, the activity of *C. erythraea* to *E. coli* was almost identical to the inhibition zones of ampicilin (Table 3). Antibiotics are molecules with selective activity towards the cells of microorganisms while essential oils are compounds with many different and, often, variable components that attack different parts of cell structures. It is extremely hard to compare zones of inhibition produced by antibiotics and bioactive compounds since they differ in nature and mechanism of the inhibition. Nevertheless, antibiotics are used as controls in any compound testing to ensure information about sensitivity of tested strains and internal control. Observed higher antimicrobial activity is presumably related to the oil principal constituents, although part of the activity could result from the presence of oil minor constituents. In general, the most active essential oils (principally composed of carvacrol, thymol, citral, eugenol and their precursors [20,21,22]) against the strains of *E. coli* are: the oil of oregano (*Origanum vulgare*), thyme (*Thymus vulgaris*), bay (*Pimenta racemosa*) and clove (*Eugenia caryophyllata*). The oil of *C. erythraea* contained thymol and carvacrol as major constituents and exhibited noticed activity against *E. coli*. Menthol is abundant in *C. erythraea* oil and the MICs demonstrated [23] that menthol is more toxic against *E. coli* than thymol. The antimicrobial activity mechanism of thymol, carvacrol and menthol is well known (alteration of membrane permeability [23]). In addition, strong antimicrobial activity of basil (*Ocimum basilicum*) oil (major constituents were linalool and 1,8-cineole) against *S. enteritidis* is known [24] and linalool was present in *C. erythraea* oil that exhibited activity against *S. enteritidis*. However, there is evidence that total essential oil is more strongly antimicrobial than is accounted for the additive effect of their major antimicrobial components; minor components appear, therefore, to play a significant role [25]. 

**Table 3:** Inhibition zones of *C. erythraea* essential oil to tested bacterial species (in mm).

	Culture	Essential oil	Antibiotics			
			Ampicilin	Norfloxacin	Ofloxacin	Tetracycline
Gram negative	*Escherichia coli*	13 ± 0.577 *	15 ± 2.828	26 ± 0	25 ± 0	20 ± 1.414
	*Pseudomonas fluorescens*	0 ± 0	**	40 ± 5.657	31 ± 0	22 ± 1.414
	*Salmonella enteritidis*	13 ± 1	24 ± 0	27 ± 0	26.5 ± 0.707	24.5 ± 0.707
Gram positive	*Bacillus cereus*	7 ± 0.693	13 ± 0.707	22 ± 0	24 ± 0.707	22 ± 0.707
	*Listeria monocytogenes*	0 ± 0	37 ± 0	30.5 ± 0.707	28.5 ± 0.707	37.5 ± 2.121
	*Staphylococcus aureus*	8 ± 0.289	45 ± 1.414	29.5 ± 3.536	29.5 ± 2.121	30.5 ± 3.536

* Average value and standard deviation of 6 discs. ** - = no inhibition.

Gram-positive bacteria demonstrated increased resistance to *C. erythraea* oil, compared to Gram negative ones. To *L. monocytogenes*, the essential oil exhibited no inhibitory effect, while weak inhibitory zones (7 and 8 mm, respectively), were observed to *B. cereus* and *S. aureus* growth. Compared to all tested species, *E. coli* and *S. enteritidis* were the most sensitive bacteria to *C. erythraea* essential oil. Of the Gram-negative bacteria, Pseudomonads, in particular *P. aeruginosa* and *P. fluorescens*, appear to be least sensitive to the action of essential oils (Table 3) [26,27].

The results (Table 3) are in contrast with previous reports indicating that Gram-positive bacteria are more susceptible to the essential oils than Gram-negative ones [28]. However, this contrast was already reported for the essential oils of three Greek *Achillea* species [29] and antimicrobial properties of the main constituents showed that caryophyllene oxide was the most efficient, followed by camphor and 1,8-cineole. The oil of *C. erythraea* contained caryophyllene oxide and camphor among minor constituents.

Kirbağ *et al.* [9] demonstrated activity of *C. erythraea* infusion to several bacterial species, where *Bacillus megaterium* exhibited highest sensitivity (13 mm) while *E. coli* and *S. aureus* showed no antibacterial activity. However, comparison with the results in Table 3 is not possible since different chemical classes of natural compounds were isolated by the plant infusion (mainly non-volatile compounds) and further tested on antibacterial activity.

## 3. Experimental

### 3.1. Plant Material, Solvents and Isolation of the Essential Oil

The aerial parts (flower, leaves and steam) of *C. erythraea* Rafn were collected in the Podravina area (Croatia) near Đurđevac and Koprivnica (voucher specimen number RH-0078). Air-dried aerial parts of *C. erythraea* were subjected to hydrodistillation for 3 h, using a Clevenger-type apparatus [30] to produce a pale yellow highly fragrant essential oil. The obtained oil was separated, dried over anhydrous sodium sulfate and stored at 4 °C until the analysis. The yield was calculated based on dry weight of the sample. The solvents pentane and diethyl ether were purchased from Kemika (HR-Zagreb) and were distilled before usage.

### 3.2. Microcolumn Oil Fractionation

The essential oil (30 μL) was fractionated on a silica gel column (4 g; 30–60 mm) and two fractions were obtained. Pentane was used for the elution of non-polar compounds and diethyl ether for the elution of polar compounds. The separation was monitored by thin layer chromatography using Kieselgel 60 aluminum-backed sheets (Merck). The obtained fractions were concentrated by fractional distillation and analysed.

### 3.3. Headspace Solid-Phase Microextraction (HS-SPME)

Two fibres (PDMS/DVB and DVB/CAR/PDMS) suitable for the extracting compounds with relatively wide range of polarities and volatilities were selected for HS-SPME after preliminary research. Other operating parameters for HS-SPME (such as extraction time and temperature) were also determined in preliminary research with respect to overall number of isolated compounds. The isolation of headspace volatiles was performed using manual SPME fibers with the layer of polydimethylsiloxane/divinylbenzene (PDMS/DVB) and divinylbenzene/carboxen/polydimethyl-siloxane (DVB/CAR/PDMS) obtained from Supelco Co (Bellefonte, PA, USA). The fibers were conditioned prior to use according to the manufacturer instructions. For HS-SPME extraction, 1 g of grounded plant material was placed in a 15 mL glass vial and hermetically sealed with PTFE/silicone septa. The vial was maintained in a water bath at 60 °C during equilibration (15 min) and extraction (45 min) and was partially submerged so that the plant material was below the water level. After sampling, the SPME fiber was withdrawn into the needle, removed from the vial, and inserted into the injector (250 °C) of the GC and GC-MS for 6 min where the extracted volatiles were thermally desorbed directly to the GC column [31]. 

### 3.4. Gas Chromatography and Mass Spectrometry

Gas chromatographic analysis was performed on an Agilent 7890 instrument (Agilent Technologies, Palo Alto, CA, USA) equipped with a flame ionization detector and an HP-5MS capillary column ((5%-phenyl)-methylpolysiloxane, Agilent J & W GC column, 30 m × 0.25 mm × 0.25 μm) or HP-FFAP column (nitroterephthalic acid modified polyethylene glycol, Agilent J &WGC column, 50 m × 0.32 mm × 0.50 μm). The compounds were identified on an Agilent Technologies 5975C mass spectrometer (MS conditions were: ionization voltage 70 eV; ion source temperature 280 °C; mass scan range: 30–300 mass units). The GC settings were as follows: the column HP-5MS (the initial oven temperature was held at 70 °C for 2 min and ramped at 3 °C min^−1^ to 200 °C.), the column HP-FFAP (the initial oven temperature was held at 70 °C for 2 min and ramped at 3 °C min^−1^ to 180 °C) and the injector temperature was maintained at 270 °C. The samples (1 μL) were injected with a split ratio of 1: 50. The carrier gas was helium at flow rate of 1.0 mL min^−1^.

The constituents were identified by comparison of their mass spectra with those stored in Wiley 275 (Wiley, New York, USA) and NIST 05 (Gaithersburg, MD, USA) libraries or with mass spectra from the literature [32,33] as well as by comparison of their retention indices with those of the literature [32,33] or with those of available authentic compounds. The retention indices were determined in relation to a homologous series of *n*-alkanes (C_8_–C_24_) under the same operating conditions. Component relative percentages were calculated based on GC peak areas without using correction factors. The component percentages (Table 1 and Table 2) were calculated as mean values from duplicate GC and GC-MS analyses.

### 3.5. Strains and Antibacterial Testing

The following bacteria (with internal codes) were obtained from Croatian National Institute of Public Health (Osijek, Croatia): *Escherichia* *coli* HZJZ32, *Bacillus* *cereus* HZJZB04, *Salmonellaenteritidis* HZJZX41 and *Listeriamonocytogenes* HZJZLL139 (isolated from patients with infection/intoxication). *Pseudomonas fluorescens* 8568 was obtained from Deutsche Sammlung von Mikroorganismen und Zellkulturen (DSMZ) GmbH (Leidnitz, Germany) and *Staphylococcus aureus* 6538 P from American Type Culture Collection (Rockville, MD, USA). Bacterial cultures were maintained on Tryptic Glucose Yeast agar (TGYE agar, Biolife, Italy) at 4 °C. Working cultures were regenerated by two successive 24 h growth cycles on TGYE agar at 37 °C, except *P. fluorescens* (incubated at 25 °C). Disk diffusion method was used to test antibacterial activity of the essential oil. By sterile swabs, standardized inoculum density (1–2 × 10^8^ CFU/mL; compared to McFarland standard 0.5) was spread over entire surface of TGYE agar plate. After drying, three blank antibiotic disks Φ 6 mm (Liofilchem, Italy) were applied on each plate. To each disk, 5 μL of essential oil (final concentration of 1 mg/mL) was transferred (5 μg/disc). After diffusion (60 minutes at 4 °C), the plates were incubated at 37 °C for 24 h (25 °C temperature for *P. fluorescens* 8568). After incubation, inhibition zone diameters were read by a ruler [34]. Antibiotics (tetracycline, ampicilin, norfloxacin and ofloxacin; Biolife, Italy) were used as positive controls. The antibiotic amounts on the discs were: ampicilin (10 μg), norfloxacin (10 μg), ofloxacin (5 μg) and tetracycline (30.55 μg).

## 4. Conclusions

The present research has provided a more detail insight into the phytochemical composition of *Centaurium erythraea* Rafn essential oil and new data for the headspace VOCs composition were obtained. Molecular mass, polarity and volatility of VOCs as well as the type of used fiber resulted in significantly different headspace/oil chemical compositions. Differences in monoterpenes, sesquiterpenes and fatty acid-derived compounds in the headspace/oil distribution were expected, but the abundance of benzene derivatives in the headspace was a surprise, particularly the high percentages of toluene and naphthalene that could be connected with a non-anthropogenic origin. The oil fractionation enabled a more detailed oil analysis (a number of compounds were found only after fractionation). Lot of similarities were found with previous reports on this oil, but different features were also noted, indicating possible moderate geographical variability with structurally similar major compounds. The essential oil showed antimicrobial activities on *Escherichia coli*, *Salmonella enteritidis*, *Staphylococcus aureus* and *Bacillus cereus*, but more microorganisms should be tested in further research for detail evaluation of its antibacterial activity.
